# Food Functional Factors in Alzheimer’s Disease Intervention: Current Research Progress

**DOI:** 10.3390/nu16233998

**Published:** 2024-11-22

**Authors:** Rong-Zu Nie, Huo-Min Luo, Ya-Ping Liu, Shuang-Shuang Wang, Yan-Jie Hou, Chen Chen, Hang Wang, Hui-Lin Lv, Xing-Yue Tao, Zhao-Hui Jing, Hao-Kun Zhang, Pei-Feng Li

**Affiliations:** 1 College of Food and Bioengineering, Zhengzhou University of Light Industry, Zhengzhou 450001, China; nierongzu@zzuli.edu.cn (R.-Z.N.); huominluo@zzuli.edu.cn (H.-M.L.); wangsh052@zzuli.edu.cn (S.-S.W.); yanjiehou@zzuli.edu.cn (Y.-J.H.); chenchen053@zzuli.edu.cn (C.C.); lvhuilin@zzuli.edu.cn (H.-L.L.);; 2 Henan Key Laboratory of Cold Chain Food Quality and Safety Control, Zhengzhou University of Light Industry, Zhengzhou 450001, China; 3 Key Laboratory of Cold Chain Food Processing and Safety Control, Ministry of Education, Zhengzhou University of Light Industry, Zhengzhou 450001, China

**Keywords:** Alzheimer’s disease, food functional factors, polyphenols, polysaccharides, unsaturated fatty acids

## Abstract

Alzheimer’s disease (AD) is a complex multifactorial neurodegenerative disease. With the escalating aging of the global population, the societal burden of this disease is increasing. Although drugs are available for the treatment of AD, their efficacy is limited and there remains no effective cure. Therefore, the identification of safe and effective prevention and treatment strategies is urgently needed. Functional factors in foods encompass a variety of natural and safe bioactive substances that show potential in the prevention and treatment of AD. However, current research focused on the use of these functional factors for the prevention and treatment of AD is in its initial stages, and a complete theoretical and application system remains to be determined. An increasing number of recent studies have found that functional factors such as polyphenols, polysaccharides, unsaturated fatty acids, melatonin, and caffeine have positive effects in delaying the progression of AD and improving cognitive function. For example, polyphenols exhibit antioxidant, anti-inflammatory, and neuroprotective effects, and polysaccharides promote neuronal growth and inhibit inflammation and oxidative stress. Additionally, unsaturated fatty acids inhibit Aβ production and Tau protein phosphorylation and reduce neuroinflammation, and melatonin has been shown to protect nerve cells and improve cognitive function by regulating mitochondrial homeostasis and autophagy. Caffeine has also been shown to inhibit inflammation and reduce neuronal damage. Future research should further explore the mechanisms of action of these functional factors and develop relevant functional foods or nutritional supplements to provide new strategies and support for the prevention and treatment of AD.

## 1. Introduction

Alzheimer’s disease is a complex multifactorial disease and the most common neurodegenerative disease worldwide [1]. Although a large number of studies have been conducted in the past few decades, the pathogenesis of AD has not yet been fully elucidated [2]. At present, the treatment of Alzheimer’s disease mainly focuses on symptom management, which has limited efficacy and cannot reverse the disease process. Therefore, the identification of safe and effective prevention and treatment methods is a widespread focus of current research. Functional factors in foods include polyphenols, polysaccharides, unsaturated fatty acids, melatonin, and caffeine. Because these are relatively safe bioactive substances, they have great potential in the regulation of human physiological functions and promotion of human health. An increasing number of recent studies have shown that food functional factors exert multiple biological activities, such as regulating the inflammatory response, anti-oxidation, and inhibiting neurotoxicity. They have also been shown to exhibit strong activity against Alzheimer’s disease [3]. However, the current research on food functional factors in the prevention and treatment of Alzheimer’s disease is still in its early stages. Therefore, a complete theoretical understanding of their effects and the best system for their application in Alzheimer’s disease remains to be determined. This article summarizes current progress in research focused on food functional factors in the prevention and treatment of Alzheimer’s disease. We also provide theoretical information and data supporting their application in Alzheimer’s disease intervention.

## 2. Research Background and Significance of Food Functional Factors in Alzheimer’s Disease

### 2.1. Alzheimer’s Disease

Alzheimer’s disease (AD) is the main type of dementia and generally develops in older individuals [4]. Its main symptom is persistent high-level neurological dysfunction that affects aspects such as memory, thinking, analysis and judgment, visuospatial recognition, emotion, personality, and general behavior [5]. The pathological characteristics of AD include cerebral cortical atrophy, β-amyloid deposition, neurofibrillary tangles caused by the hyperphosphorylation of Tau proteins, as well as a substantial reduction in the number of memory neurons and the formation of senile plaques [6]. Although a variety of drugs are currently used for the treatment of AD, their efficacy is not ideal.

**Figure 1:**
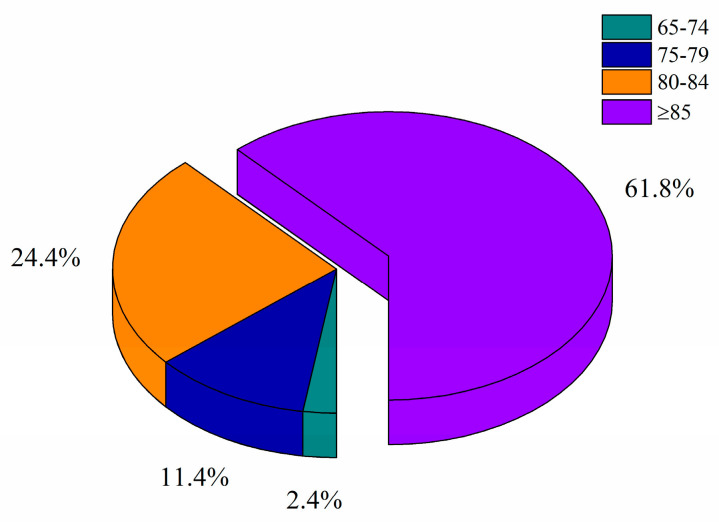
The proportion of Alzheimer’s disease patients over 65 years by age group.

As the disease progresses, AD patients may gradually lose their ability to perform daily activities, placing a heavy burden on families and society [7]. Studies have confirmed that the prevalence of AD increases with age, in particular in people over 65 years of age (Figure 1) [8,9]. The risk of developing the disease doubles for every 6.1 years of age increase [10]. In addition, the global economic losses caused by AD reach hundreds of billions of dollars every year. As the global population ages, this number is expected to continue to rise in the coming decades. Therefore, as AD becomes a major problem plaguing global economic and social development, research on its pathogenesis and treatment strategies has increasingly received widespread attention from the academic community.

### 2.2. Food Functional Factors

Food functional factors are natural chemical components present in food that exert specific physiological activities and can produce beneficial regulatory effects on human health [11]. Recently, an increasing number of studies have shown that food functional factors can effectively interfere with the occurrence and development of AD. When used as nutritional supplements or raw materials for functional foods, they have been shown to significantly improve cognitive ability, behavioral performance, and quality of life in AD patients, playing an important role in preventing and alleviating AD [12]. In addition, food functional factors also have the advantages of safety, economy, and widespread availability. Therefore, they have received widespread attention from the academic community.

Studies have found that polyphenols, polysaccharides, unsaturated fatty acids, melatonin, and caffeine have strong antioxidant, anti-inflammatory, and anticoagulant activities [13]. For example, tea polyphenols, soy isoflavones, resveratrol, and seaweed polysaccharides are widely reported as food functional factors that can intervene in AD (Table 1). Through the study of food functional factors, we can gain a deeper understanding of the pathogenesis and prevention of AD as well as determine a theoretical basis for the development of new therapeutic drugs and intervention measures. Conducting relevant research on the use of food functional factors in AD allows for the exploration of new AD prevention and treatment strategies. This research also promotes the development of the functional food industry, increases public health awareness, and further improves the health of the elderly. Therefore, research on the use of food functional factors in AD holds important theoretical and practical significance.

## 3. Research Status of Food Functional Factors in Alzheimer’s Disease

### 3.1. Polyphenols

Polyphenols are a class of natural organic compounds widely distributed in the plant kingdom that carry more than two phenolic hydroxyl groups on their benzene ring structures. These compounds are mainly concentrated in fruits, vegetables, tea, wine, and chocolate, and include catechins, flavonoids, and phenolic acids [33]. Polyphenol compounds exert antioxidant, anti-inflammatory, neuroprotective, and amyloid protein deposition-regulating effects, and therefore may have significant inhibitory effects in AD [33].

The catechin polyphenol compound Epigallocatechin gallate (EGCG) is one of the main components of tea polyphenols. Its biological activity in intervening in AD has attracted widespread attention [34]. For example, EGCG has been shown to inhibit Aβ-induced SH-SY5Y neuronal cell damage, enhance cell activity, and reduce apoptosis in a dose-dependent manner [35]. In a subsequent mechanistic study, it was found that this effect played a role in the down-regulation of GRP78, CHOP, and cleaved-caspase-12 and -3. Additionally, EGCG reduced the cytotoxicity caused by tunicamycin (TM) and thapsigargin (TG), both of which are ER stress activators. The senescence-accelerated mouse prone-8 (SAMP-8) mouse is an accelerated aging model that was commonly used to study Alzheimer’s disease. In the SAMP-8 mouse model, the level of malondialdehyde (MDA) in mouse brain hippocampal tissue decreased to a level similar to that in normal mice following EGCG intake [36]. This suggests that EGCG may protect neurons in the hippocampus by reducing lipid peroxidation and thus reducing oxidative stress. In addition, EGCG intervention in APP/PS1 transgenic rats, a commonly used model for studying Alzheimer’s disease, further showed that the expression levels of TLR4 and NF-κB were reduced after EGCG treatment, indicating that EGCG reduced the inflammatory response and decreased neurotoxicity [37].

Soybean isoflavones (SIFs) are secondary metabolites formed during the growth of cereals, beans, and other plants. SIFs exhibit antioxidant and anti-inflammatory effects and also inhibit aggregation of the Aβ amyloid peptide [38]. Studies have shown that intake of SIFs significantly promoted nerve cell proliferation in the hippocampus and increased dendrite length in AD model mice, suggesting SIFs may have a positive effect on neurodegenerative diseases [39]. The CaM-CaMKIV signaling pathway plays an important role in cell signal transduction. Cai Biao et al. found that SIFs down-regulated the CaM-CaMKIV signaling pathway and the expression of multiple downstream proteins to inhibit the production of Aβ, thereby exerting a protective effect on PC12 cells [40]. In addition, SIFs also reduced the level of Aβ production by reducing the synthesis of amyloid precursor protein (APP) and directly regulating the expression of α, β, and γ secretases [39]. Moreover, SIFs have the ability to directly destroy the hydrogen bonds and hydrophobic bonds of Aβ in amyloid peptides and can also chelate metal ions to effectively inhibit the formation of toxic Aβ oligomers [41]. These effects can reduce the accumulation of Aβ in the brain, thereby reducing the risk of AD.

Curcumin is a natural polyphenol compound extracted from the rhizome of turmeric. It is abundant in curry. It has shown great potential in the prevention and treatment of AD due to its biological activities, such as enhancing synaptic plasticity, improving memory function, and increasing autophagy [42]. Researchers used 4-week-old C57BL/6 mice, divided them into groups, and orally administered low-dose curcumin (0.4 mg/kg) daily to observe changes in behavioral and brain physiological indicators [43]. The results showed that low-dose curcumin enhanced synaptic plasticity, promoted neurogenesis in the hippocampus by increasing neuronal proliferation and differentiation, and improved memory function [43]. Sorrenti et al. [17] studied the effect of curcumin pretreatment on acute neuroinflammation in mice induced by lipopolysaccharide (LPS). After two consecutive days of oral pretreatment with 50 mg/kg curcumin, high-dose LPS was injected intraperitoneally. The results showed that a single high-dose intraperitoneal injection of LPS increased the number of activated microglia in the cerebral cortex of mice and increased the mRNA expression of pro-inflammatory mediators such as TNF-α, IL-1β, NLRP3 inflammasome, IL-6, and COX-2, while early intervention with curcumin attenuated the acute inflammatory response induced by LPS.

Several studies have demonstrated that standardized Ginkgo biloba extract (EGb761) has antioxidant, neuroprotective, and cognitive improvement effects. Its components include 24% flavonoid glycosides and 6% terpene lactones. The former is a polyphenolic compound [44]. One study selected volunteers aged 50 to 65 years old with a certain degree of subjective memory impairment and average or slightly below average cognitive function. The volunteers were randomly divided into an EGb761 group (240 mg EGb761 per day) and a placebo group (placebo with the same appearance) for 56 ± 4 days. Cognitive function was assessed using a task switching paradigm and a Go-NoGo task [45]. The results showed that after treatment with EGb761, cognitive flexibility in the task switching paradigm was improved, and there was also an improvement trend in controlling the trade-off between reaction speed and accuracy in the Go-NoGo task [45]. In addition, oxidative stress often leads to mitochondrial dysfunction. EGb761 can inhibit the release of mitochondrial cytochrome c, up-regulate the expression of the anti-apoptotic protein Bcl-2, down-regulate the expression of the pro-apoptotic protein Bax, and reduce the activity of caspase-9 and caspase-3. This reduces oxidative stress-induced apoptosis and protects neurons from damage [18].

One study used near-infrared brain imaging technology (functional Near-Infrared Spectroscopy, fNIRS) to explore the effect of polyphenols on AD [46]. This technology uses the difference in the absorption rate of near-infrared light of different wavelengths under different oxygen-carrying conditions of hemoglobin to reflect the functional state of the cerebral cortex. One study including 36 subjects performed neuropsychological scales and task-based functional near-infrared tests at baseline and follow-up [46]. The results showed that polyphenol intervention improved the cognitive function and brain blood oxygen levels of AD patients [46]. Other studies have shown that polyphenols can also improve the cognitive ability and quality of life of AD patients by regulating estrogen levels, protecting neurons, and inhibiting the inflammatory response [47,48]. In addition, polyphenols have also been shown to increase antioxidant enzyme activity, enhance the antioxidant capacity of PC12 neuronal cells, relieve Ca^2+^ overload, and reduce glutamate-induced PC12 neuronal cell damage [49].

### 3.2. Polysaccharides

Polysaccharides, which have been shown to exert multiple biological activities, are carbohydrates composed of more than 10 monosaccharides [50]. Recently, polysaccharides have attracted widespread attention in the biomedical field due to their potential to improve cognitive function. Their mechanism of action involves promoting neuronal growth, inhibiting inflammation and oxidative stress, and increasing cerebral blood flow in the SAMP-8 mouse model [50]. These mechanisms make polysaccharides a potential functional factor that can effectively prevent and alleviate cognitive dysfunction.

*Lycium barbarum* polysaccharide 1 (LBP1), a polysaccharide extracted from wolfberry, has been shown to exhibit anti-inflammatory, antioxidant, and neuroprotective effects [22]. Researchers constructed an AD animal model using APP/PS1 transgenic mice and tested cognitive function using the Morris Water Maze (MWM) test, which evaluates spatial learning and memory, and then further explored its mechanism of influence on cognitive function [22]. The results showed that LBP1 reduced Aβ levels and amyloid plaque accumulation, promoted neurogenesis and neural progenitor cell (NPC) proliferation and restored hippocampal synaptic plasticity in APP/PS1 mice. This resulted in improved cognitive function [22].

*Ganoderma lucidum* Polysaccharide (GLP) is a secondary metabolite produced by the mycelium of the genus *Ganoderma* fungus of the family Polyporaceae and is distributed in the mycelium and fruiting bodies [51]. GLP has been shown to exhibit multiple biological activities such as neuroprotection, immunomodulation, and anti-aging, which may aid in the prevention and delay the occurrence and development of AD [23]. NPCs can differentiate into various types of neurons and play an important role in maintaining and repairing nervous system function [52]. Studies have found that oral intake of GLP can stimulate NPC proliferation, thereby promoting the generation of new neurons and alleviating cognitive deficits in transgenic AD mice [52]. Further studies have shown that GLP can enhance the activation of fibroblast growth factor receptor 1 (FGFR1) and its downstream signaling pathways, including extracellular signal-regulated kinase (ERK) and protein kinase B (Akt) [52]. When FGFR1 is inhibited, GLP-induced NPC proliferation and activation of downstream signaling pathways are also effectively blocked [52]. Therefore, GLP may act as a regeneration-promoting factor, enhance FGFR signaling, and promote neurogenesis in the absence of growth factors, thus alleviating the cognitive decline associated with neurodegenerative diseases [52].

### 3.3. Unsaturated Fatty Acids

Fish oil is a rich source of omega-3 polyunsaturated fatty acids including α-linolenic acid (ALA), eicosapentaenoic acid (EPA), and docosahexaenoic acid (DHA). These fatty acids have been shown to exhibit biological activities such as anti-inflammatory effects and lowering blood lipids [53].

β-Secretase and γ-Secretase are two enzymes in the Aβ peptide production pathway that are involved in the metabolic process of APP [54]. Studies have shown that, after treatment with EPA and DHA, the content and activity of β-Secretase and γ-Secretase in cell culture supernatant and the serum of aged mouse models decreased, inhibiting the production of Aβ [55]. Tau protein is a key protein in the pathogenesis of AD. Tau protein molecules in the brains of healthy people usually contain two to three phosphate groups, while Tau protein molecules in AD patients often contain five or more [56]. Hyperphosphorylation can cause changes in the structure and function of Tau proteins, thereby affecting the normal function of neurons, ultimately causing neuronal death and cognitive dysfunction [57]. Studies have shown that DHA can reverse Tau protein hyperphosphorylation by inhibiting the increase in JNK activity, thereby protecting the recognition and cognitive abilities of mice and potentially providing a new strategy for the prevention and treatment of AD [58]. Similarly to DHA, EPA also inhibits the hyperphosphorylation of Tau proteins. EPA promotes the activation of the Akt signaling pathway in neurons, thereby inhibiting the activity of the GSK-3β enzyme and reducing the phosphorylation of Tau proteins [59].

Omega-3 polyunsaturated fatty acids have also been shown to inhibit the inflammatory response [24]. Neuroinflammation is one of the multiple factors leading to the occurrence of AD [60]. Microglia and astrocytes are the main inflammation-related cells in the central nervous system (CNS) [24]. Studies at the cellular level found that supplementation with EPA and DHA significantly improved neuroinflammation in AD patients and reduced levels of inflammatory biomarkers and pro-inflammatory cytokines [61].

Among the apolipoprotein E (ApoE) alleles, the ApoE4 gene is highly correlated with late-onset AD. Individuals carrying this gene have a 3 to 15 times higher risk of developing AD than those without the gene [62]. Xie Tianzhi used ApoE4 transgenic mice to establish an AD animal model and explored the effects of omega-3 polyunsaturated fatty acids on lipid metabolism disorders and cognitive dysfunction following long-term feeding of a high-fat diet [25]. The results showed that omega-3 polyunsaturated fatty acids alleviated brain lipid metabolism disorders and suppressed the gene expression of *APP* and *Bace1* in the brains of ApoE4 transgenic mice. This resulted in reduced Aβ deposition. In addition, the findings revealed that omega-3 polyunsaturated fatty acids not only significantly reduced the oxidative damage in ApoE4 transgenic mice, but also inhibited activation of the TLR4/NF-κB signaling pathway, leading to decreased expression of downstream pro-inflammatory cytokines such as TNF-α, IL-1β and IL-6. This resulted in mitigation of neuroinflammation and improved cognitive dysfunction [25].

### 3.4. Melatonin

Melatonin (MLT) is a hormone that is synthesized by the pineal gland and regulates sleep [63]. In addition to acting on biological rhythms, it also has multiple biological activities such as scavenging free radicals, anti-oxidation, anti-inflammation, regulating neurotransmitters, and immunosuppression [26]. The research on MLT in AD is a key issue in current academic circles. The literature has shown that AD patients experience obvious circadian rhythm disorders, which is associated with the secretion of MLT [64]. An increasing number of studies have shown that reduced sleep increases the production of Aβ, leading to cognitive decline [27]. The above studies revealed that MLT not only exerts important maintenance and regulatory functions under normal physiological conditions, but also has the potential to delay the occurrence and development of AD [65].

Death-associated protein kinase 1 (DAPK1) can affect the pathological process of AD by regulating Aβ and neuronal autophagy [66]. Studies have shown that in AD patients, DAPK1 expression levels increased significantly while MLT levels decreased [66]. Further research found that the combination of MLT and DAPK1 inhibitors can synergistically reduce the accumulation and phosphorylation of Tau proteins and promote neurite outgrowth and microtubule structure assembly in C57BL/6 mouse embryonic cortical neurons [67]. The above research results confirm the key role of MLT in regulating Tau protein phosphorylation, providing a potential new method of AD intervention.

Brain-derived neurotrophic factor (BDNF) plays a crucial role in synaptic plasticity, learning, memory, and neurogenesis [68]. BDNF signaling can stimulate long-term potentiation of hippocampal synapses, thereby improving spatial memory [69]. Studies have shown that BDNF expression is decreased in AD patients, especially in brain regions that are closely related to learning and memory, such as the hippocampus [70]. Acetylcholine is an important neurotransmitter that is essential to cognitive function and memory. Donepezil (DON) is an acetylcholinesterase inhibitor. By inhibiting the action of acetylcholinesterase, DON can improve AD symptoms by increasing acetylcholine levels in the brain [69]. Studies have found that the combination of MLT and DON can synergistically increase the expression of the BDNF gene and reverse the decreased BDNF protein levels in the hippocampus of AD mouse models [70]. cAMP response element binding protein (CREB) is a nuclear transcription factor that plays a key role in the transcription of BDNF. Reduction in CREB phosphorylation leads to the down-regulation of BDNF levels, which in turn affects memory [63]. In addition, CREB is also involved in intracellular signal transduction and regulates the circadian rhythm of long-term memory effectors in the hippocampus [71]. Studies have shown that MLT can improve spatial memory in AD animal models by regulating expression of the BDNF and CREB1 genes in the hippocampus of AD mice [70]. Therefore, MLT has great potential to alleviate AD memory impairment.

Mitochondria are not only important sites for energy production, but they also bear the important tasks of sensing cell stress stimuli and determining cell fate. They are important organelles for maintaining the normal structure and physiological function of neurons [72]. Mitochondrial homeostasis is crucial for reducing cellular damage due to oxidative stress. Mitochondrial homeostasis imbalance is involved in the pathophysiological processes of a variety of central nervous system diseases including neurodegenerative diseases, brain trauma, brain tumors, and stroke [73]. In terms of mitochondrial biogenesis, studies have shown that MLT can stimulate the generation of mitochondria [28]. Zhang Bin et al. found that MLT increased mitochondrial biogenesis in C57BL/6J mouse embryonic cortical neurons following H_2_O_2_-induced damage, increased mitochondrial membrane potential, increased the expression of mitochondrial fission protein Drp1, and reduced the expression of mitochondrial fusion protein Mfn2 [28]. In this way, MLT promoted mitochondrial biogenesis and played a role in maintaining neuronal mitochondrial homeostasis following H_2_O_2_-induced damage [28]. We found that melatonin modulated mitochondrial homeostasis by phosphorylating AMPK in C57BL/6J mouse embryonic cortical neurons following H_2_O_2_-induced damage. When neurons were pretreated with the AMPK inhibitor WZ4003, the effect of melatonin on regulating mitochondrial homeostasis was notably weakened. Thus, it is plausible to speculate that, following neuronal H_2_O_2_ injury, the AMPK signaling pathway played a crucial role in melatonin- induced maintenance of mitochondrial homeostasis in neurons [28]. In addition, by increasing the number and quality of mitochondria, MLT helped maintain energy balance in the cell, supported the normal function of neurons, and reversed mitochondrial dysfunction [74,75]. Mitochondrial autophagy is responsible for clearing damaged mitochondria and preventing the release of apoptotic factors and ROS [76]. MLT promotes the fusion of mitochondrial autophagosomes and lysosomes to restore mitochondrial autophagy, aiding in the clearing of dysfunctional mitochondria, reducing oxidative stress, and enhancing mitochondrial energy metabolism and antioxidant capacity. Through this mechanism, MLT thereby protects neurons from damage, reduces Aβ pathological deposition, and improves cognitive function [77].

### 3.5. Caffeine

Caffeine is a central nervous system stimulant extracted from coffee and tea. It is a xanthine alkaloid compound with potential neuroprotective effects [78]. Caffeine can play a positive role in the intervention of cognitive decline in people with early signs of AD (mild cognitive impairment, MCI), especially in terms of executive function, planning, self-control, and attention [79].

Caffeine has been shown to inhibit the inflammatory response and reduce neuronal damage. Caffeine intake has been shown to inhibit the production of inflammatory-related cytokines in male C57BL/6J mice such as interleukin-1β [29]. Caffeine exerts anti-inflammatory effects by regulating the expression of inflammation-related genes and affecting cell signal transduction [29]. ICAM-1 is a cell adhesion molecule closely involved in the inflammatory response, and its expression is increased in many inflammatory diseases [80]. Through the use of specific inhibitors and siRNA knockout experiments, it was further found that caffeine inhibits the activation of the p38 MAPK signaling pathway, thereby inhibiting the expression of ICAM-1 [81]. One study explored the molecular mechanisms by which caffeine alleviates neuroinflammation through the p38 MAPK/MK2 signaling axis. The experiments used qPCR and siRNA knockout technology to confirm that caffeine reduced neuronal damage and synaptic dysregulation by inhibiting p38 MAPK [82]. Taken together, these studies reveal that caffeine may be used as a potential anti-inflammatory substance with great potential for alleviating inflammation-related neurodegenerative diseases.

Caffeine has also been shown to increase the expression of antioxidant enzymes such as superoxide dismutase (SOD) and catalase (CAT), which aid in the removal of free radicals and reduce oxidative stress-induced damage to neurons [30]. Studies have shown that caffeine can promote the expression of antioxidant enzymes by activating the Nrf2 pathway, thereby enhancing cellular defense mechanisms against free radical damage [83]. Nrf2 is a key transcription factor in the regulation of cellular defense mechanisms against free radical damage [31]. In addition to Nrf2, adenosine receptors (ADORs) play an important role in resisting oxidative stress and neuroinflammation [31]. Currently, there are four known ADORs: A1R, A2AR, A2BR, and A3R. Among these, A2AR is a key receptor for resisting neuronal damage, ischemia, and hypoxia [31]. Interestingly, caffeine has been shown to reduce oxidative stress by regulating the expression of A2AR in cell models [31]. Studies have also shown that caffeine exerts an inhibitory effect on neuronal apoptosis through A1R [84]. The above research results confirm that caffeine can have an antioxidant effect and show that it can protect cells from oxidative stress-induced damage at the molecular level.

Caffeine can also inhibit neuronal apoptosis and protect neurons from damage through a variety of other mechanisms [32]. Caffeine inhibits Ca^2+^ influx and increases the expression of Ca^2+^ pumps, thereby regulating Ca^2+^ balance and protecting neurons from damage caused by Ca^2+^ overload, a key factor in neuronal apoptosis [85]. Furthermore, caffeine can change the content of neurotransmitters, thereby affecting signal transduction and apoptosis of neurons [86]. In addition, caffeine may also affect the structure and function of neuronal cell membranes, further affecting neuronal survival and apoptosis [86].

### 3.6. Other Food Functional Factors

**Figure 2:**
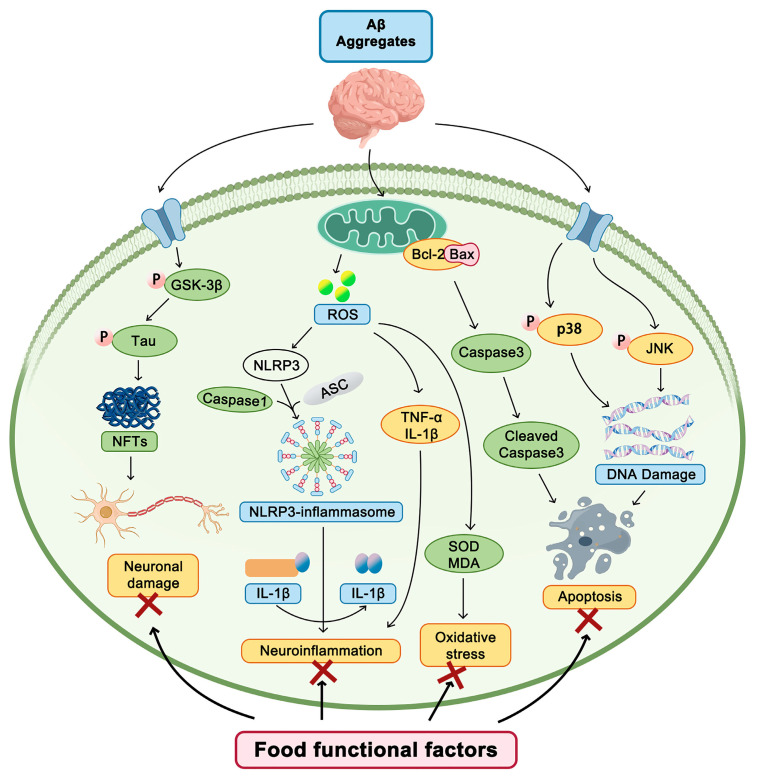
Molecular mechanisms of food functional factors in Alzheimer’s disease.

The molecular mechanisms of the above-mentioned food functional factors in AD involve multiple aspects, as outlined in Figure 2. Other functional factors, such as active peptides and active proteins, oligosaccharides, sugar alcohols, saponins, vitamins, and minerals have also exhibited strong efficacy in preventing and intervening in AD. The mechanisms of action of these functional factors include regulating the immune response, scavenging free radicals, protecting neurons, and promoting nerve regeneration.

Active peptides and proteins such as soy protein, whey protein, and glutathione have been shown to have exert antioxidant and anti-inflammatory effects. By employing PC12 cells and the zebrafish model, previous studies have shown that these ingredients can protect nerve cells from oxidative damage and inhibit the inflammatory response [87]. The biologically active ingredients in whey protein, such as lactoferrin and immunoglobulins, have been shown to exhibit neuroprotective, antioxidant, anti-inflammatory, and immunomodulatory effects in male Wistar rats [88]. Studies have shown that whey protein hydrolysate (WPH), which contains the pentapeptide leucine-aspartate-isoleucine-glutamine-lysine (LDIQK), significantly reduced p-Tau levels (a key marker of tauopathy) and Bcl2 associated X (BAX) (a pro-apoptosis factor). In addition, the protein levels of BDNF and B-cell lymphoma 2 (Bcl2, an anti-apoptotic factor) were enhanced. Moreover, WPH was found to activate the Nrf2/HO-1 signaling pathway, which is involved in the antioxidant response. Therefore, the WPH containing LDIQK demonstrated neuroprotective effects against H_2_O_2_-induced cell damage in mouse hippocampus-derived HT22 neuronal cells. This suggests that WPH, or its active peptide, LDIQK, may serve as a potential edible agent for improving cognitive dysfunction [89]. Glutathione is an important polypeptide antioxidant that has been shown to effectively scavenge free radicals and other oxidants in nerve cells, reducing oxidative stress-induced damage. Glutathione has also shown anti-inflammatory and antioxidant effects by lowering serum IL-6, insulin, testosterone, C-reactive protein (CRP), and MDA levels in female rats, thereby reducing the degree and duration of the inflammatory response [90]. In addition, glutathione has also been shown to have a positive effect in AD by promoting neuronal metabolism and energy production, improving the repair and regeneration capacity of brain neurons [91,92].

Oligosaccharides and sugar alcohols have shown multiple biological activities such as improving intestinal microecological balance, enhancing immunity, and anti-aging properties [93,94]. Oligosaccharides are carbohydrate chains composed of 2–10 sugar molecules; examples include maltooligosaccharide, galactooligosaccharide, and oligofructose. [94]. Studies have shown that intestinal health is closely related to the risk of AD. Oligosaccharides are broken down and utilized by beneficial bacteria, such as bifidobacteria, in the intestines. They can also inhibit the reproduction of harmful bacteria and reduce intestinal inflammation and bacterial translocation, thus potentially reducing the occurrence and development of AD [95,96,97]. Sugar alcohols are used as low-calorie natural sweeteners and mainly include compounds such as sorbitol, maltitol, and xylitol [98]. Interestingly, studies have found that increased blood sugar levels in humans are accompanied by an increased risk of AD [99,100]. Because sugar alcohols have a low GI value (Glycemic Index), they do not induce increased blood sugar levels. They have also been shown to exhibit anti-inflammatory and antioxidant effects. These properties may aid in protecting nerve cells from damage [98,101].

Saponins are a class of glycosidic compounds naturally occurring in plants whose aglycones can include triterpenes or spirosterane compounds. Saponins are mainly distributed in terrestrial higher plants and also exist in small amounts in marine organisms such as starfish and sea cucumbers [102]. Many Chinese herbal medicines such as ginseng, polygala, platycodon, licorice, rhizoma anemarrhenae, and bupleurum contain saponins as the main active ingredient [102]. The saponin ginsenoside Rg1 is the main active ingredient in ginseng. In APP/PS1 transgenic mice, Rg1 exhibited anti-inflammatory and antioxidant effects, and inhibited oxidative stress and apoptosis, thus having a neuroprotective effect [103]. Rg1 has also been shown to reduce the production of ROS and prevent LPS-induced neuronal apoptosis in sepsis-associated encephalopathy (SAE) mice by up-regulating the expression levels of Nrf2 and HO-1 [104]. Rg1 also alleviated LPS-induced neuronal damage by inhibiting the expression of NLRP1 inflammasome in HT22 mouse hippocampal neuronal cells [105]. Studies have also shown that Rg1 can alleviate LPS-induced neuroinflammation in ICR mice, reduce learning and memory dysfunction, and decrease neuronal damage through inhibition of NOX2-mediated ROS production and increasing Ca^2+^ ion concentration [106].

Finally, vitamins and minerals such as vitamin C, vitamin E, and magnesium have been shown to exhibit antioxidant and neuroprotective functions. Studies have shown that these ingredients can have a regulatory effect on the neurotransmitter system and are important to the normal functioning of the immune system [107]. For example, vitamin C is a highly active antioxidant that can scavenge free radicals and exert a protective effect on nerve cells [108,109]. Vitamin C is also involved in the synthesis and metabolism of neurotransmitters, suggesting it may play a role in improving cognitive function [110].

## 4. Conclusions and Limitations

Food functional factors are an emerging potential means of preventing and alleviating the symptoms of AD. These factors have the advantages of safety, economy, and ease of access. As discussed in this review, food functional factors have been shown to exhibit numerous positive effects on AD in cell, animal, and human models through various mechanisms such as anti-inflammation, anti-oxidation, regulation of lipid metabolism, and promotion of nerve growth and repair. However, the current study has several limitations. I: Current research on the use of food functional factors in AD is limited by a lack of unified research methods and the current limited understanding of the mechanisms of action. In addition, due to the complexity of the etiology and pathological mechanisms of AD, experimental evidence on the effects of single food functional factors on AD remains inadequate. Therefore, future study is required using improved research methods and more in-depth clarification of the molecular mechanisms of food functional factors in AD. II: Existing studies mostly focus on short-term interventions, and the long-term effects have not been fully verified. There remains a gap between animal experiments and clinical studies. How to effectively convert the experimental results of animal models into intervention methods suitable for the human population is still a challenge. Future studies should focus on this problem. III: The effects of a variety of food functional factors in AD intervention are crucial. Different functional factors show multi-faceted intervention potential through anti-oxidation, anti-inflammation, improving neuroprotection, regulating lipid metabolism, and promoting neuroregeneration. Future studies should explore the synergistic effects of these functional factors by studying the interactions between different factors and their synergistic effects in multi-mechanism and multi-pathway interventions. This will lead to more accurate and effective nutritional intervention strategies based on a combination of multifunctional factors. At the same time, further clarifying the molecular mechanisms of these food functional factors can provide a theoretical basis for the targeted application of specific functional factors and provide new strategies for the prevention and treatment of Alzheimer’s disease.

## Figures and Tables

**Table 1:** Intervention using Functional Factors in AD.

Food Functional Factors	Food Sources	Mechanism of Action	Molecular Pathway	References
Tea polyphenols	Green tea, black tea, oolong tea	Antioxidant, anti-inflammatory, neuroprotective, regulate neurotransmitter levels, and inhibit the formation of amyloid peptide	Endoplasmic reticulum (ER) stress pathway TLR4/NF-κB signaling pathway	[14,15]
Soy Isoflavones	Soybean and its products	Anti-inflammatory effects, reduce oxidative stress	CaM-CaMKIV signaling pathway	[16]
Curcumin	Turmeric Rhizome	Anti-inflammatory, antioxidant, enhance synaptic plasticity, improve memory function, and increase autophagy	NLRP3 inflammasome signaling pathway	[17]
Ginkgo biloba extract	Ginkgo leaf	Antioxidant, neuroprotective, and cognitive improvement effects	Bcl-2/Bax/Caspase-3 signaling pathway	[18]
Resveratrol	Red wine, grapes and peanuts	Activate longevity proteins involved in neuroprotection, antioxidant effects, inhibit inflammatory response, reduce Aβ aggregation and improve mitochondrial function	SIRT1/AMPK signaling pathway	[19,20]
Seaweed polysaccharide	Kelp, laver, agar	Neuroprotective effects, immunomodulatory effects, reduce oxidative stress	BDNF-TrkB-ERK signaling pathway	[21]
*Lycium barbarum* polysaccharide	Wolfberry	Anti-inflammatory, antioxidant, and neuroprotective	Aβ peptide production pathway	[22]
*Ganoderma lucidum* polysaccharide	*Ganoderma*	Nerve protection, immune regulation, anti-aging	FGFR/ERK signaling pathway	[23]
Omega-3 polyunsaturated fatty acids	Olive oil, camellia oil, nuts	Regulate lipid metabolism, relieve inflammation and oxidative stress, and reduce Aβ deposition	Aβ peptide production pathway TLR4/NF-κB signaling pathway	[24,25]
Melatonin	Walnuts, cherries, oats	Regulate neuroinflammation, regulate sleep, anti-oxidative stress, improve mitochondrial function	BDNF/CREB signaling pathway AMPK signaling pathway	[26,27,28]
Caffeine	Coffee, cocoa beans, tea leaves	Antagonize adenosine receptors, reduce inflammation, resist oxidation, enhance autophagy, protect neurons	p38 MAPK signaling pathway Nrf2 signaling pathway	[29,30,31,32]
